# Harnessing neutrophil plasticity for HCC immunotherapy

**DOI:** 10.1042/EBC20220245

**Published:** 2023-09-28

**Authors:** Erik Ramon-Gil, Daniel Geh, Jack Leslie

**Affiliations:** 1 Newcastle Fibrosis Research Group, Biosciences Institute, Faculty of Medical Sciences, Newcastle University, Newcastle Upon Tyne, U.K.; 2 The Newcastle University Centre for Cancer, Newcastle University, Newcastle Upon Tyne, U.K.

**Keywords:** hepatocellular carcinoma, immunotherapy, neutrophils

## Abstract

Neutrophils, until recently, have typically been considered a homogeneous population of terminally differentiated cells with highly conserved functions in homeostasis and disease. In hepatocellular carcinoma (HCC), tumour-associated neutrophils (TANs) are predominantly thought to play a pro-tumour role, promoting all aspects of HCC development and progression. Recent developments in single-cell technologies are now providing a greater insight and appreciation for the level of cellular heterogeneity displayed by TANs in the HCC tumour microenvironment, which we have been able to correlate with other TAN signatures in datasets for gastric cancer, pancreatic ductal adenocarcinoma (PDAC) and non-small cell lung cancer (NSCLC). TANs with classical pro-tumour signatures have been identified as well as neutrophils primed for anti-tumour functions that, if activated and expanded, could become a potential therapeutic approach. In recent years, therapeutic targeting of neutrophils in HCC has been typically focused on impairing the recruitment of pro-tumour neutrophils. This has now been coupled with immune checkpoint blockade with the aim to stimulate lymphocyte-mediated anti-tumour immunity whilst impairing neutrophil-mediated immunosuppression. As a result, neutrophil-directed therapies are now entering clinical trials for HCC. Pharmacological targeting along with *ex vivo* reprogramming of neutrophils in HCC patients is, however, in its infancy and a greater understanding of neutrophil heterogeneity, with a view to exploit it, may pave the way for improved immunotherapy outcomes. This review will cover the recent developments in our understanding of neutrophil heterogeneity in HCC and how neutrophils can be harnessed to improve HCC immunotherapy.

## Introduction

**Figure 1:**
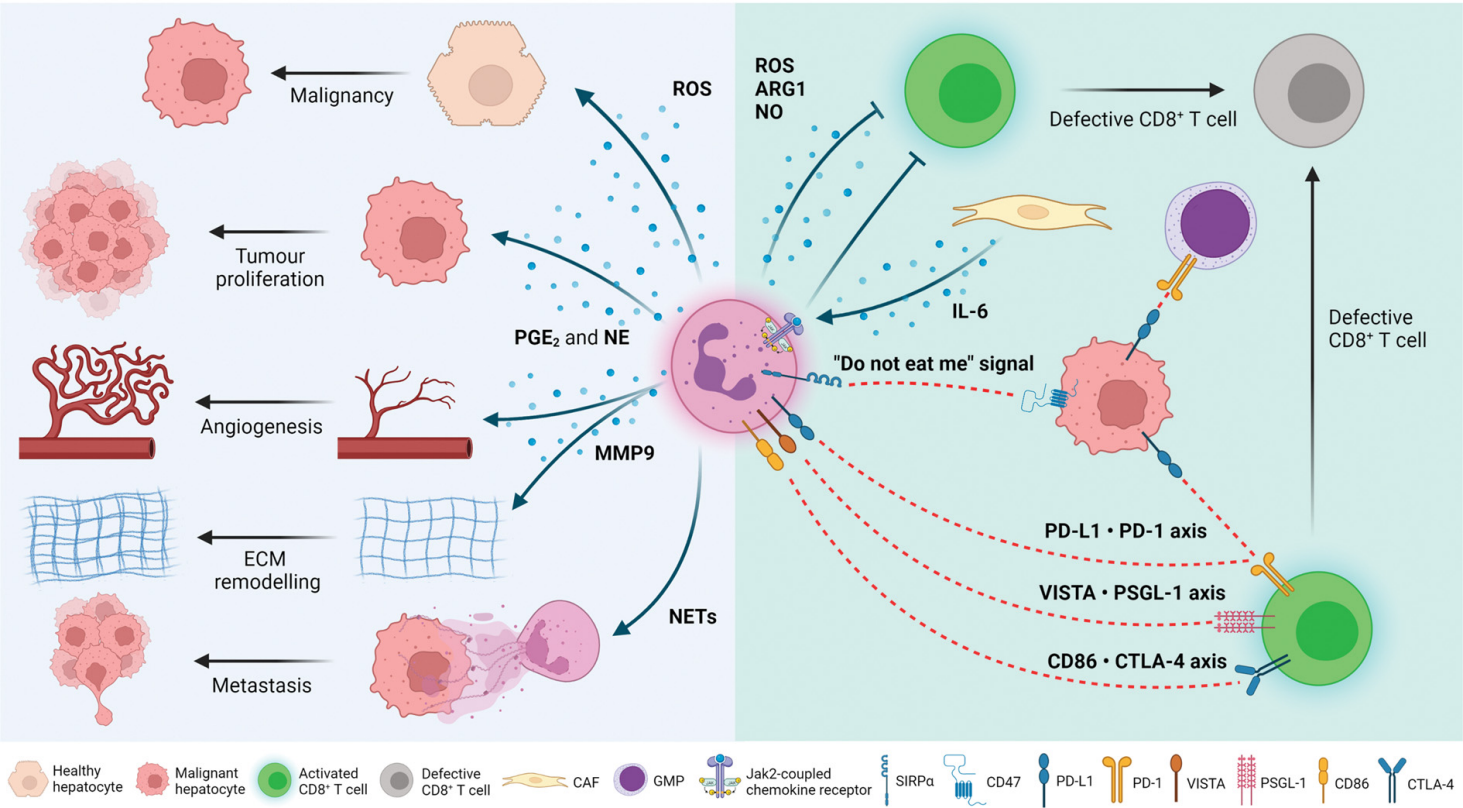
Pro-tumour mechanisms of tumour-associated neutrophils *Direct effects of neutrophils on cancer cells*: Neutrophils release a plethora of bioactive molecules that drive cancer development. Neutrophils secrete reactive oxygen species (ROS) which, through DNA damage, can drive malignant transformation of hepatocytes. They also secrete prostaglandin E_2_ (PGE_2_) and neutrophil elastase (NE), which can induce tumour cell proliferation; as well as matrix metalloproteinase 9 (MMP9), which promotes angiogenesis and extracellular matrix (ECM) remodelling in the tumour. Furthermore, release of neutrophil extracellular traps (NETs) promotes metastatic seeding of tumour cells in distal organs. Tumour cells express CD47 which, when bound to its ligand, signal regulatory protein α (SIRPα) found on the surface of neutrophils, impairs their phagocytic capacity; a process commonly known as the ‘do not eat me’ signal. A proportion of tumour cells also express the programmed death-ligand 1 (PD-L1) which, in contact with programmed cell death protein 1 (PD-1) in the surface of CD8^+^ T cells and granulocyte–monocyte progenitors (GMPs), suppresses their anti-tumour functions. *Effects of neutrophils on the immune system*: Neutrophils emit ROS, arginase 1 (ARG1) and nitric oxide (NO), which can immunosuppress cytotoxic CD8^+^ T cells. Moreover, cancer-associated fibroblasts (CAFs) secrete interleukin 6 (IL-6) that is recognised by JAK2-coupled chemokine receptors on the surface of neutrophils, also immunosuppressing T cells. Finally, neutrophils express PD-L1, V-type immunoglobulin domain-containing suppressor of T-cell activation (VISTA) and CD86 which, in contact with their respective receptors/ligands on CD8^+^ T cells, can impair their cytotoxic ability. Figure created with BioRender.com

Primary liver cancer is a leading cause of cancer-related death and morbidity, with 905,667 cases and 830,180 related deaths in 2020 [1]. Worryingly, the incidence of primary liver cancer is expected to increase up to 55% by 2040 [2]. Hepatocellular carcinoma (HCC) is the most common form of primary liver cancer making it a significant public health concern. HCC typically develops on the background of chronic liver disease and cirrhosis of various aetiologies. This makes delivering effective therapy challenging due to aetiology-specific immunosuppressive tumour microenvironments and patient frailty due to underlying liver disease and co-morbidities in typically elderly patients. Although there are curative treatments available for early-stage HCC, the majority of HCCs are diagnosed at an advanced stage with limited palliative treatments available and a generally poor prognosis. The IMBrave150 phase III clinical trial resulted in the approval of the immunotherapy combination of Atezolizumab (anti-PD-L1) plus Bevacizumab (anti-VEGF) as a first-line systemic treatment for advanced disease and the first significant change in HCC patient treatment for over a decade [3]. Whilst this immunotherapy combination is highly effective in a subset of patients (one-third responders), the majority of patients fail to clinically respond [4]. Failure to respond to immunotherapy is typically associated with an immunosuppressive HCC tumour microenvironment (TME). Currently approved immunotherapies target immune-inhibitory receptors (e.g. PD-1 and CTLA-4) on T lymphocytes, or their ligands (e.g. PD-L1), aiming to activate T-lymphocyte cytotoxicity and anti-tumour immunity [5]. New therapies targeting immunosuppressive cells in the TME beyond T lymphocytes, alone or in combination with current immunotherapies, might be more effective in a subgroup of HCC patients. Neutrophils are one such target. In health, neutrophils comprise 50–70% of all circulating leukocytes and are the first effector cells to arrive at sites of infection, inflammation and tissue damage [6]. Advancement in single-cell RNA sequencing (scRNAseq) technologies has finally enabled analysis of tissue-derived neutrophils at the single-cell level, allowing us to understand their previously unappreciated plasticity and heterogeneity in chronic disease and cancer. Tumour-associated neutrophils (TANs), through their effector functions; phagocytosis, degranulation, release of neutrophil extracellular traps (NETs) and antigen presentation, have been implicated in a broad range of pro- and anti-tumour activities in HCC, from promoting tumour cell proliferation, immunosuppression, metastasis and angiogenesis to direct tumour killing and activation of anti-tumour immunity, respectively [7–9], and their pro-tumour roles are summarised in Figure 1. Given our improved understanding of the level of cellular heterogeneity and plasticity neutrophils are capable of, future work will need to determine whether the broad range of pro- and anti-tumour roles previously identified for neutrophils are present in either all or discrete populations of TANs, which may have therapeutic implications.

This review will cover recent advances in our understanding of neutrophil function and heterogeneity in HCC and discuss how this heterogeneous population of neutrophils may impact the TME and their cellular plasticity can be harnessed to improve immunotherapy in HCC.

## Neutrophil heterogeneity in granulopoiesis

Phenotypical and functional heterogeneity in myeloid cells is well established for monocyte and macrophage populations and assures diverse functions that are required to maintain homeostasis, respond to infection and resolve inflammation [10]. On the other hand, neutrophils are traditionally considered as a homogeneous population of terminally differentiated cells with highly conserved function. Recent advances in single-cell technologies are challenging this view and are confirming that human and murine neutrophils are a heterogeneous population of cells that display a range of maturation and polarisation states. This heterogeneity is governed by a multitude of factors including tissue of residence, physiologic state (e.g. homeostasis, inflammation and cancer), circadian rhythms and neutrophil cellular age as well as obesity, sex, tobacco consumption and whole organism aging [11–14].

Initial transcriptomic studies in 2020 [15–17] defined up to eight different neutrophil populations in mice and human, ranging from granulocyte-macrophage progenitor (GMP) precursors to mature circulating neutrophils, with identified clusters unified using the following nomenclature: G0, G1, G2, G3, G4, G5a, G5b and G5c [17]. These clusters have distinct maturation states, a predominant tissue of residence (bone marrow, spleen and peripheral blood), and are present under homeostasis and perturbed during pathogenic infection. G0, G1, G2, G3 and G4 clusters align to GMP, proNeu, preNeu, immNeu and mNeu, respectively, and reside predominantly in the bone marrow. Peripheral blood contained three main neutrophil subsets; G5a, G5b and G5c, which had typical mature neutrophil nuclear morphology. Given the distinct transcriptional signatures of circulating neutrophils, which could not be explained by cellular age, mechanical stress, or insults, it has been proposed that these subsets may be pre-programmed to perform specific homeostatic functions.

Our group and others have suggested that neutrophil maturation plays a critical role in determining their phenotype and functionality. Fundamental to this, is the recently proposed ‘neutrotime paradigm’ which aims to provide a framework to understand and describe heterogeneity in neutrophil populations. In this model, neutrophils, rather than existing in discrete maturation states as described previously, follow a developmental continuum, called the ‘neutrotime’ [18]. As neutrophils progress along this continuum and encounter a stimulus they will begin to acquire a phenotype (or polarisation state), that is the product of both time (e.g. maturation stage) and environmental cues (sequence of signals encountered). This mechanism provides an opportunity to generate a highly diverse neutrophil repertoire without a requirement for committed developmental branches or subsets [18]. Neutrophils recruited to sites of inflammation and infection, typically emerge from the mature end of the neutrotime spectrum, containing a full complement of anti-microbial granule proteins allowing them to perform their primary function, clearing pathogens. However, during chronic disease and cancer, where distortions in granulopoiesis are observed, emergence of neutrophils earlier along the neutrotime continuum is common with circulating neutrophils appearing relatively immature. As a result, these cells are phenotypically and functionally distinct from their mature counterparts which have encountered similar environmental cues. Alterations in granulopoiesis in cancer has also led to reports of extended neutrophil lifespan [19], possibly in excess of 5 days for circulating human neutrophils [20], providing further time for phenotype alterations.

## Neutrophil heterogeneity in cancer: the age of single-cell technologies

For over a decade, neutrophil heterogeneity in cancer has been dichotomously described with circulating neutrophils labelled based on their density as either high/normal- or low-density neutrophils (HDNs/NDNs and LDNs, respectively) and TANs labelled based on either their identified pro- or anti-tumour functions [21]. Emerging evidence is now confirming that this simple binary characterisation is not sufficient to describe the broad range of neutrophil phenotypes and proposed widespread functionality.

Single-cell transcriptomics has revolutionised our understanding of the tumour microenvironment, providing a wealth of data on nearly all cell types at single-cell resolution. However, until recently, the majority of scRNAseq studies failed to recover a significant number of neutrophils for downstream analysis; this is a feature also prominent in other single-cell analyses performed in solid organs [22–26]. Reasons behind this include the fragile nature of neutrophils during isolation and the relative low abundance of RNA compared with other cell types. Progress, however, has been made, with recent first reports of neutrophil heterogeneity in liver cancer [27] and other solid tumours such as gastric cancer [28], pancreatic ductal adenocarcinoma (PDAC) [29] and non-small cell lung cancer (NSCLC) [30].

## Neutrophil heterogeneity in HCC

**Figure 2:**
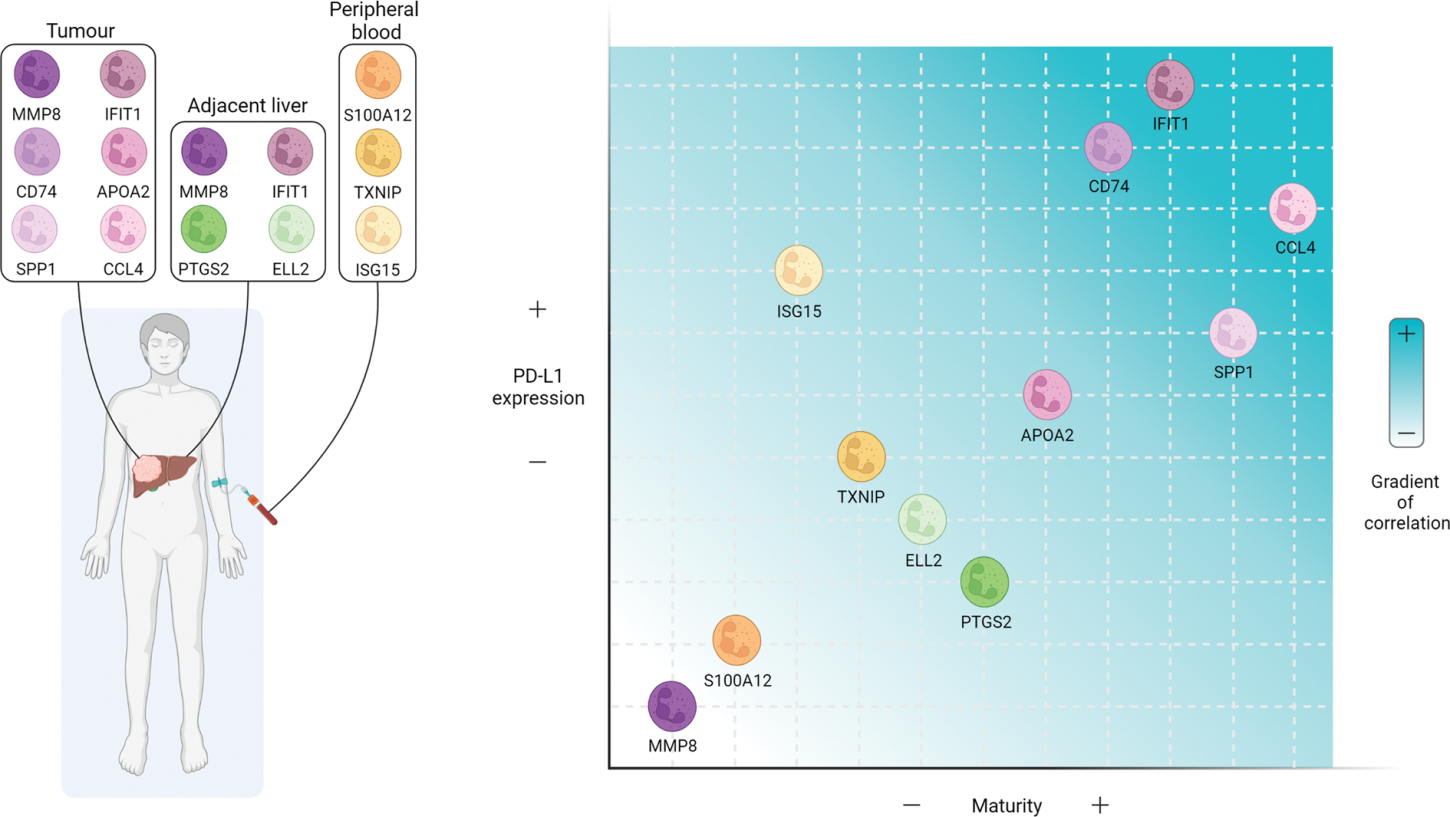
Neutrophil heterogeneity in liver cancer Up to 11 different neutrophil phenotypes have been described in human HCC by scRNAseq [27]. These phenotypes have different predominant tissues of residence, including tumour, adjacent liver and peripheral blood. Moreover, they display a range of maturity stages and PD-L1 expression levels that show a positive correlation. Figure created with BioRender.com

The study of neutrophil subtypes in HCC is still in its infancy. Despite many single-cell transcriptomic analyses of HCC having been performed [31–38] the first description of significant neutrophil heterogeneity in HCC patients and preclinical models was made only in 2022 [27], highlighting the technical difficulty of neutrophil heterogeneity analysis and its emergence as a novel area of research in HCC. Xue et al. analysed 160 samples of 124 treatment-naïve liver cancer patients, of which 100 samples were from 79 HCC patients, comprising a mixture of viral (HBV and HCV) and non-viral HCC. It must be noted that this study was performed on resection samples and as such may not be representative of patients with more advanced HCC, where resections cannot be performed. As such presence of the following neutrophil/TANs will need to be confirmed in biopsy tissue from patients with advanced disease. The authors identified 11 transcriptionally distinct neutrophil clusters in HCC which they termed: Neu_01_MMP8, Neu_02_S100A12, Neu_03_ISG15, Neu_04_TXNIP, Neu_05_ELL2, Neu_06_PTGS2, Neu_07_APOA2, Neu_08_CD74, Neu_09_IFIT1, Neu_10_SPP1 and Neu_11_CCL4. The S100A12^+^, ISG15^+^ and TXNIP^+^ subtypes are found in peripheral blood, the ELL2^+^ and PTGS2^+^ mainly in adjacent liver and the MMP8^+^, APOA2^+^, CD74^+^, IFIT1^+^, SPP1^+^ and CCL4^+^ predominantly located in the tumour (Figure 2). These neutrophil clusters were conserved in a genetically engineered mouse model (GEMM), called pTMC (Myc-Δ90Ctnnb1), which is driven by β-catenin and develops HCC tumours. These neutrophil clusters exist along a maturation gradient ranging from the most immature cells present in peripheral blood, the intermediate present in adjacent liver and the most mature cells present in the tumour, with the exception of the MMP8^+^ cluster, found in the adjacent liver and tumour, having the most immature phenotype (Figure 2). Divergence of neutrophil polarising states arising from both mature and immature neutrophils provides further validity to the ‘neutrotime paradigm’ as an effective model to explain neutrophil heterogeneity in homeostasis and disease.

Understanding the biological significance of each of the TAN clusters in HCC development, how they regulate the TME and potential effects on immunotherapy responses are key questions that need to be answered. PD-L1 expression appears to be largely related to maturation in both human and mouse HCC and may provide some insight into the potential pro- or anti-tumour functions of these TANs (Figure 2). In line with this, enrichment of high PD-L1 expressing IFIT1^+^, SPP1^+^ and CCL4^+^ TANs (Figure 2) in HCC tumours correlated with a poorer prognosis indicating a pro-tumour role for these clusters [27]. It is tempting to speculate these HCC tumours would respond to immune checkpoint blockade (anti-PD-L1/anti-PD-1) and those enriched with PD-L1 low TANs will not respond. However, our results suggest that mature TANs express high levels of PD-L1 and correlate with immunotherapy resistance in HCC [39]. Further research needs to be conducted in order to understand this paradox, controlling for the expression of PD-L1 in other cell types including cancer cells, other myeloid cells like macrophages and dendritic cells, and stromal cells, as well as the molecular and immune subtype classification of the tumour, which is known to predict immune checkpoint inhibition response.

**Figure 3:**
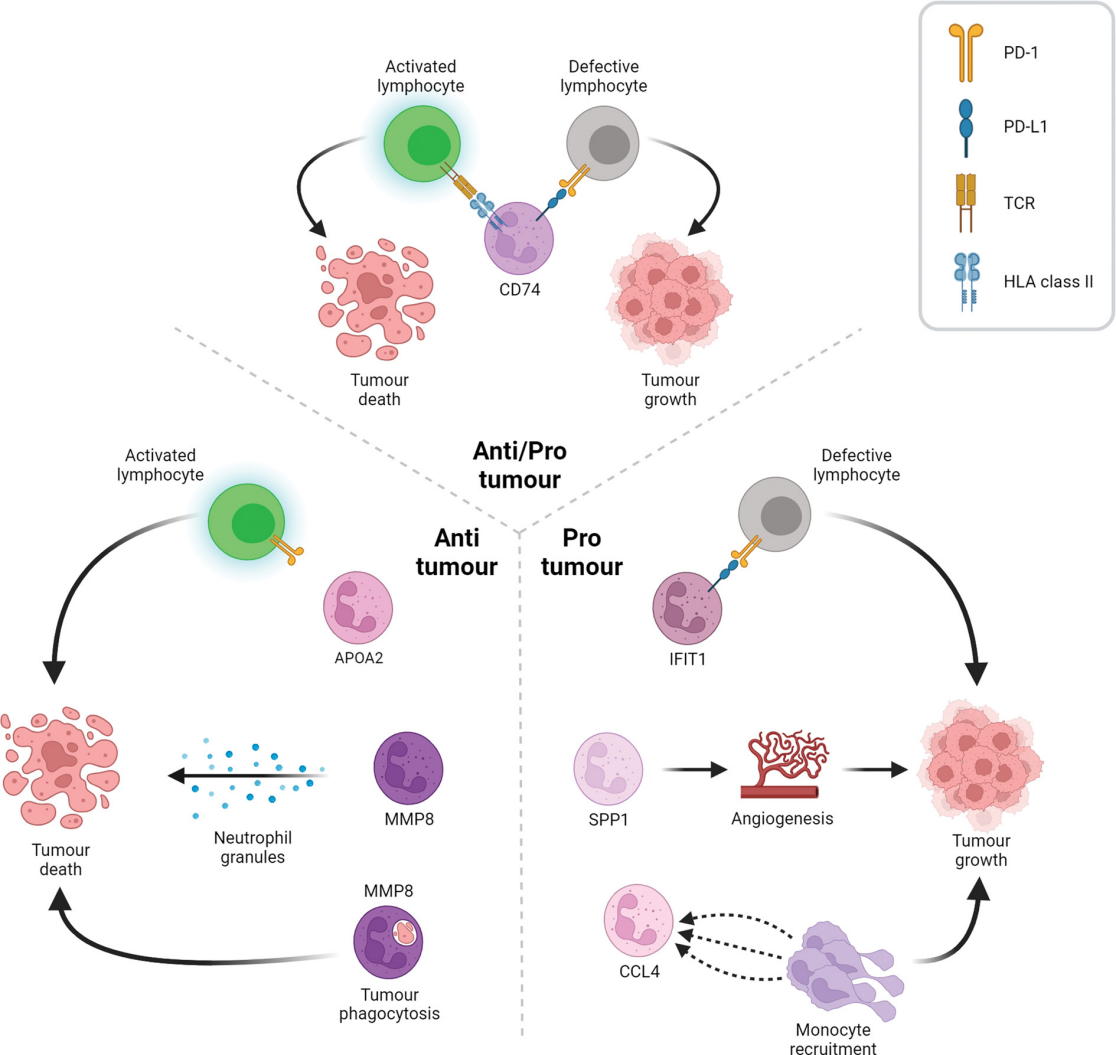
Role of tumour-associated neutrophils in the HCC microenvironment The six TANs found in the scRNAseq dataset are predicted to play diverse roles in the HCC TME. On the left side, the APOA2 subtype expresses low levels of PD-L1, which avoids the suppression of T lymphocytes that act against the tumour. Moreover, MMP8 shows an elevated secretory profile of intracellular enzyme-loaded granules that results in cell toxicity for the tumour. On the right side, the IFIT1 subtype that, although characterised for an intense interferon signalling, possesses the highest PD-L1 expression, which turns the T lymphocytes defective upon binding to their PD-1 receptor. Furthermore, the SPP1 subtype, which expresses pro-angiogenic factors, enhances angiogenic programs in the TME, resulting in tumour fuelling. The third pro-tumour TAN, the CCL4 subtype, secretes myeloid chemoattractant peptides that potentiates the infiltration of immunosuppressive monocytes to the TME. Finally, on the upper side, the CD74 subtype, which is able to activate T lymphocyte-dependant tumour death programs through its antigen-presenting activity, at the same time that possesses the second most elevated PD-L1 expression, which suppresses T lymphocytes and reinvigorates tumour growth. Figure created with BioRender.com

The six HCC TAN clusters; MMP8^+^, APOA2^+^, CD74^+^, IFIT1^+^, SPP1^+^ and CCL4^+^ can be characterised by predicted function and role in tumour development. The IFIT1^+^, SPP1^+^ and CCL4^+^ TANs were identified as pro-tumour, were associated with a poorer prognosis and had the highest levels of PD-L1 expression. These TANs were proposed to exert their pro-tumour functions by different mechanisms. IFIT1^+^ TANs were enriched for genes associated with interferon (IFN) signalling and pathways of type I interferon signalling and response to interferon gamma [40–51]. In addition, IFIT1^+^ TANs showed the highest expression of PD-L1 and could be the major population of PD-L1^+^ TANs that inhibits the cytotoxic capacity of CD8^+^ T cells, as such, these were termed interferon-stimulated immunosuppressive TANs. SPP1^+^ TANs displayed a gene expression signature similar to pro-angiogenic tumour-associated macrophages (TAMs) [52,53] and were therefore predicted to play a key role in tumour angiogenesis, thus termed angiogenic TANs. The CCL4^+^ TANs were enriched for chemokine secretion and predicted to promote the recruitment of immunosuppressive myeloid cells [54–56] and thus termed myeloid chemokine-secreting TANs (Figure 3). CD74^+^ TANs showed high expression of CD74, although protein expression of CD74 in neutrophils has not been reported yet in the bibliography [57], and other MHC-associated genes [58–62] and were termed antigen-presenting TANs. These are predicted to play a role in antigen presentation, which has classically been linked to anti-tumour functions in other cancers like lung cancer [63,64]. However, although they correlate with a better prognosis in patients, their relatively high expression of PD-L1 makes unclear whether these are likely to play a pro- or anti-tumour role (Figure 3). Finally, the MMP8^+^ and APOA2^+^ TANs expressed lower levels of PD-L1, correlated with a better prognosis and were therefore predicted to play an anti-tumour role in HCC (Figure 3). MMP8^+^ TANs were enriched for gene signatures associated with classical neutrophil properties including azurophil and gelatinase granules' secretion, neutrophil activation and phagocytosis, which have all been proposed as potential anti-tumour mechanisms [39,65–68] and were termed immature TANs. The APOA2^+^ neutrophils were enriched for metabolic pathways such as triglyceride metabolism and regulation of steroid metabolism [69–74] and thus termed hepatic lipid-associated TANs (Figure 3) and hepatic lipid-associated neutrophils or LANs, in the tumour and adjacent liver, respectively. Interestingly, LANs resemble hepatic lipid-associated macrophages (LAMs), which help maintain lipid metabolism in healthy liver and suggestive to hepatoprotective potential, suppressing HCC development [75], implying these neutrophils/TANs may play a similar role in HCC.

**Figure 4:**
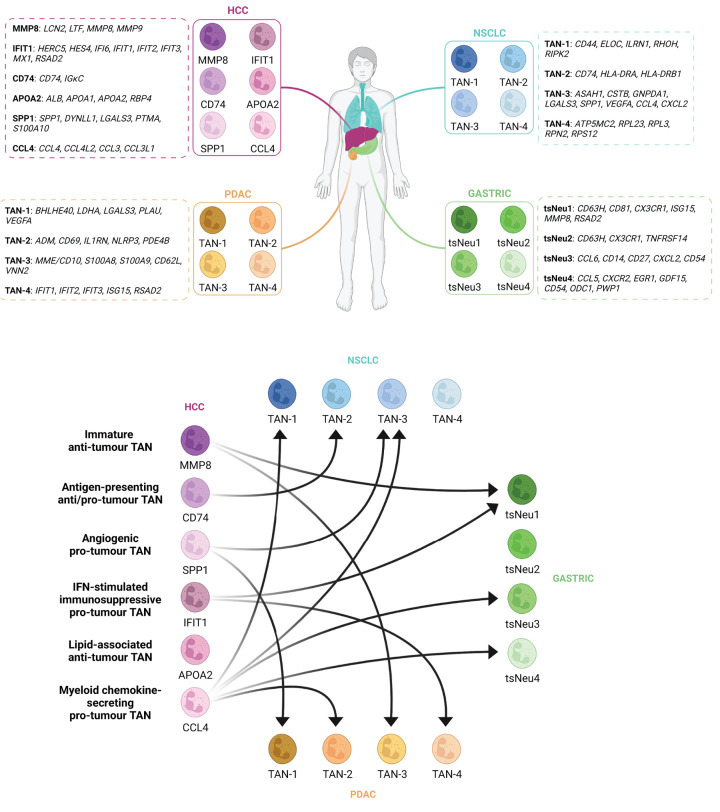
Functional correlation of neutrophil subtypes between multiple cancers The upper part of the figure shows the different neutrophil subtypes found in HCC [27], gastric cancer [28], PDAC [29] and NSCLC [30] by scRNAseq along with the list of the most differentially expressed genes for each one. The bottom part of the figure represents the functional correlation between the diverse neutrophil subtypes in the four cancers studied. Hence, the MMP8 subtype in HCC, which possess an immature anti-tumour profile, correlates with tsNeu1 gastric cancer and TAN-3 PDAC. Secondly, the CD74 subtype in HCC, with an antigen-presenting anti/pro-tumour potential, correlates with TAN-2 NSCLC. Thirdly, the SPP1 subtype in HCC, characterised for secreting pro-angiogenic factors, correlates with TAN-3 NSCLC and TAN-1 PDAC. Fourthly, the IFIT1 subtype in HCC, with an interferon-stimulated immunosuppressive pro-tumour profile, correlates with tsNeu1 gastric cancer and TAN-4 PDAC. Fifthly, the APOA2 subtype in HCC, with a lipid-associated anti-tumour role remains specific for HCC, as TAN-4 NSCLC and tsNeu2 gastric cancer do. Finally, the CCL4 subtype in HCC, with a myeloid chemokine-secreting pro-tumour profile, correlates with TAN-1 and TAN-3 NSCLC, tsNeu3 and tsNeu4 gastric cancer and TAN-2 PDAC. Figure created with BioRender.com

Interestingly, the HCC TAN clusters [27] can be identified in scRNAseq analysis of other cancers including gastric cancer [28], pancreatic ductal adenocarcinoma (PDAC) [29] and non-small cell lung cancer (NSCLC) [30] by comparison of most differentially expressed marker genes, with the exception of APOA2^+^ lipid-associated TANs which so far appear to be specific to the liver. Several TAN subtypes were found to be present in at least three of the four scRNAseq neutrophil analyses. Firstly, pro-tumour TAN subtypes were identified in all four datasets. Angiogenic TANs were identified in PDAC (TAN-1), NSCLC (TAN-3) and HCC (SPP1^+^). IFN-stimulated immunosuppressive TANs were identified in PDAC (TAN-4), gastric cancer (tsNeu1) and HCC (IFIT1^+^). Interestingly, variations of myeloid chemokine-secreting TANs were identified in all four cancers including two types identified in gastric cancer (tsNeu3 and tsNeu4), NSCLC (TAN-3) and HCC (CCL4^+^), all of which have elevated levels of myeloid chemokines. Similar TANs identified in NSCLC (TAN-1) and PDAC (TAN-2) are predicted to be pro-inflammatory and are therefore likely to contribute to myeloid cell recruitment. Antigen-presenting TANs were identified in NSCLC (TAN-2) and HCC (CD74^+^). Finally, immature TANs that are likely to play anti-tumour roles were identified in PDAC (TAN-3), gastric cancer (tsNeu1) and HCC (MMP8^+^). Interestingly, tsNeu1 TANs identified in gastric cancer expressed genes associated with immature and IFN-stimulated immunosuppressive TANs, as such, it is difficult to hypothesise a role for these TANs in tumour progression. Given our current understanding of neutrophil heterogeneity across multiple cancers, therapies that expand intratumoural MMP8^+^ and APOA2^+^ TANs whilst supressing IFIT1^+^, SPP1^+^ and CCL4^+^ TANs may be effective in improving immunotherapy responses in HCC (Figure 4).

## Harnessing neutrophils for immunotherapy in HCC

TANs are predominantly thought to be immunosuppressive in HCC favouring tumour progression [7], with preclinical pan-neutrophil depletion experiments shown to be effective in reducing tumour burden in rodents [27,76]. Therapies leading to neutropenia such as traditional chemotherapy agents, are typically poorly tolerated in HCC patients, who often have cirrhosis and portal hypertension. This coupled with the high risk of severe bacterial infection makes complete clearance of neutrophils in the absence of direct tumour cytotoxic effects not an appealing therapeutic strategy. Given the recent advances in our understanding of neutrophil heterogeneity in the HCC tumour microenvironment it may be possible to exploit their cellular plasticity allowing for the specific targeting or reprogramming of TANs with pro-tumour functions, without affecting the circulating pool of functional anti-microbial neutrophils.

## Targeting neutrophils: combination therapies and the ‘recruitment paradox’

To date, most neutrophil-based therapies have aimed to either suppress recruitment of TANs or modulate their immunosuppressive nature to elicit an anti-tumour response. Recruitment and modulation of neutrophils in HCC and their associated targeted therapies have been reviewed elsewhere in detail [7]. In this section, we will discuss recent advances in our understanding of combination immunotherapies in HCC, how this impacts neutrophil recruitment and tumour burden, and how therapies that elicit neutrophil-based anti-tumour responses can improve anti-PD-1 immunotherapy.

**Figure 5:**
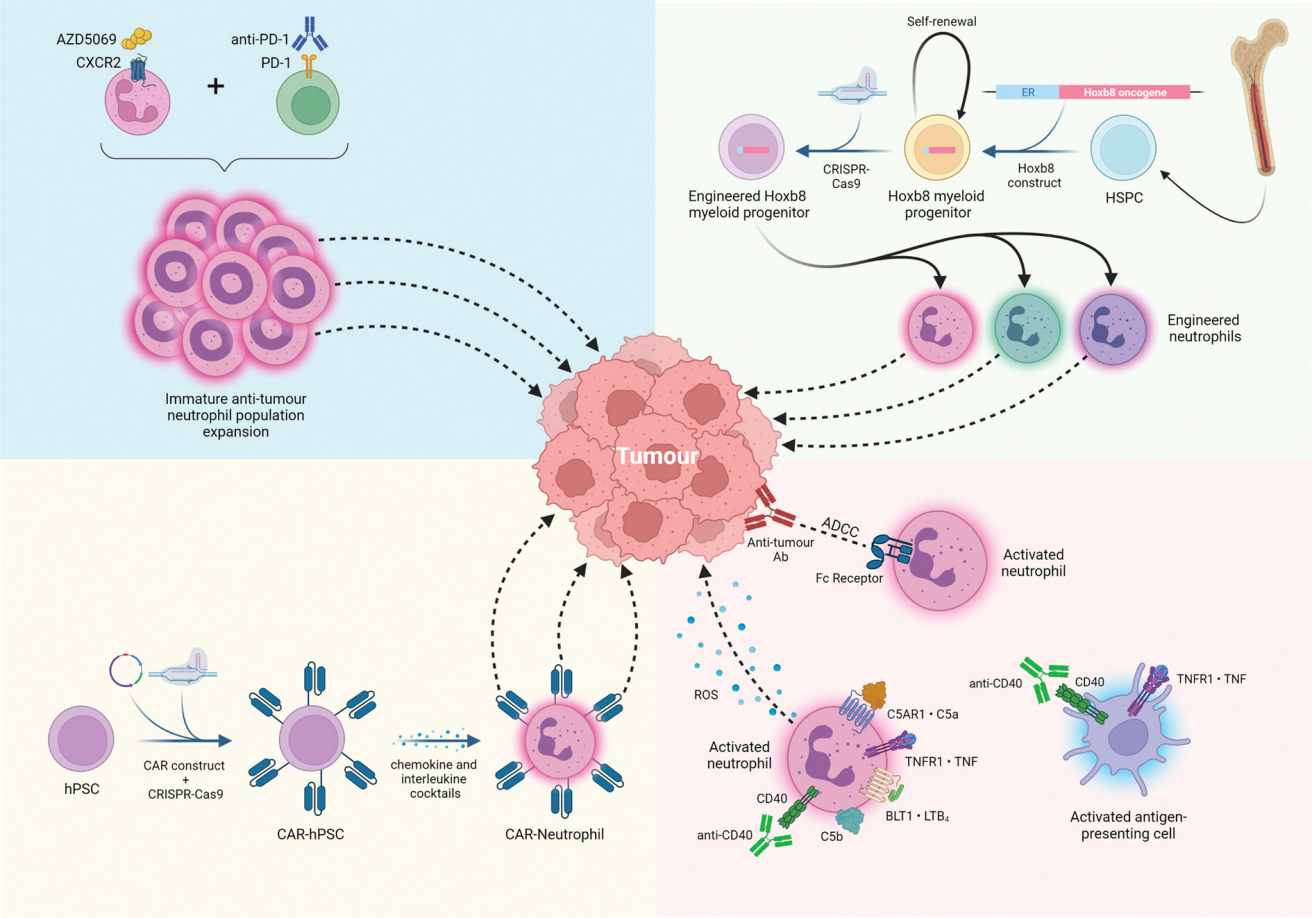
Current clinical and preclinical neutrophil therapies in cancer Top left corner: the combination of a CXCR2 small molecule inhibitor (AZD5069) plus anti-PD-1 expanded an immature anti-tumour neutrophil population, resulting in a reduction in tumour burden and an increase in survival [39]; clinical trial ongoing targeting these axes in advanced HCC [89]. Top right corner: Hoxb8 myeloid progenitors provide a continuous pool of neutrophils for genetic modification, enabling the identification of novel neutrophil-specific therapeutic targets to improve immunotherapy. Bottom left corner: the recent development of human CAR-neutrophils from human pluripotent stem cells (hPSCs) showed high efficacy in a model of glioblastoma [96] and may be suitable for use in HCC. Bottom right corner: Further immunotherapy approaches, combining TNF and monoclonal antibodies anti-CD40 and anti-tumour-associated antigen, result in a high infiltration of activated anti-tumour neutrophils and antigen-presenting cells’ activation [91]. Figure created with BioRender.com

The effectiveness of targeting neutrophil chemokine receptors in combination with immune checkpoint blockade, chemotherapy and hormone therapy has been reported for nearly a decade, in various cancers, including pancreas, lung, prostate, breast, bladder, nasopharyngeal carcinoma, ovary [77–85] and, in 2022, the liver [39]. These studies aimed to limit the recruitment of pro-tumour immunosuppressive neutrophils, largely through targeting the neutrophil specific chemokine receptor CXCR2, which would allow a robust anti-tumour response to be generated. In GEMMs of intrahepatic cholangiocarcinoma (ICC) [86], Haining Liu and colleagues demonstrated that co-inhibition of both CXCR2 and METTL1, a protein directly involved in the expression of the soluble chemo attractants CXCL5/CXCL8, in combination with anti-PD-1, achieved a complete response, with a survival rate of 100% during 60 days of tracking [87]. This therapeutic response was associated with a significant reduction in infiltrating tumour neutrophils (referred to as polymorphonuclear myeloid-derived suppressor cells). Interestingly, however, we have demonstrated that the pharmacological inhibition of both CXCR2 and PD-1 sensitises immunotherapy-resistant non-alcoholic steatohepatitis-associated HCC (NASH-HCC) mouse models to immune checkpoint blockade, resulting in an influx of intratumoural neutrophils that promote CD8^+^ T cell and CD103^+^XCR1^+^ cDC1 dendritic cell activation, leading to a decrease in tumour burden (Figure 5) [39]. These recruited neutrophils were of a lactoferrin-positive immature anti-tumour phenotype and were organised in immune hubs, which we propose act to establish and maintain anti-tumour responses in the NASH-HCC immunotherapy-resistant niche [39]. This ‘recruitment paradox’ of combination immunotherapies in NASH-HCC, we suggest, could be the result of several mechanisms. By their very nature, mature neutrophils express the highest levels of surface CXCR2 and would therefore be preferentially repressed by a CXCR2 antagonist, leading to the potential accumulation of immature neutrophils. Furthermore, synergy of CXCR2 and anti-PD-1 inhibition led to the expansion of an intratumoural immature neutrophil population originating from either a neutrophil progenitor (NeP) or tumour-seeded hematopoietic stem cell. This therapeutic synergy is also responsible for the acquisition of an anti-tumour phenotype characterised by elevated neutrophil activation and phagocytosis as well as antigen presentation capability. Indeed, a remarkably similar population of tumour-associated immature neutrophils (MMP8^+^ subtype) was identified by scRNAseq analysis and expressed genes associated with the early neutrotime signature [27]. Interestingly, this immature neutrophil population had the lowest expression of PD-L1 (CD274) among all neutrophil subsets identified and could, if expanded, represent an interesting mechanism to stimulate anti-tumour immunity. Therefore, it is clear that the therapeutic combination of CXCR2 inhibition and immune checkpoint immunotherapy overcomes a significant clinical problem faced with anti-PD-1 resistance in NASH-HCC [88] and a phase I/II clinical trial for advanced HCC [89] is currently underway targeting the CXCR2 and PD-L1/PD-1 axes.

Given that neutrophils display a range of anti-tumour functions, we propose that due to their cellular plasticity it may be possible to reprogram TANs to promote anti-tumour immunity and that this may not necessarily be specific to targeting a chemokine receptor as we have demonstrated. Esteban-Fabró and colleagues showed that combining anti-PD-1 therapy with Cabozantinib, a small molecule tyrosine kinase inhibitor largely selective for c-Met and VEGFR2, increased anti-tumour efficacy compared with monotherapies [90]. This therapeutic effect was associated with a neutrophil-based immune response, which, when used to stratify human HCC patients, represented those with favourable molecular and clinical features [90]. In melanoma models, immunotherapies combining tumour necrosis factor (TNF), anti-CD40 and anti-tumour-associated antigen antibody have also been shown to polarise neutrophils to an anti-tumour phenotype promoting tumour clearance via ROS secretion and improved antibody-dependant cellular cytotoxicity (ADCC). This provides further evidence that neutrophils have a substantial cellular plasticity which could be therapeutically exploited in HCC (Figure 5) [91].

Due to our recent improvements in the understanding of intratumoural neutrophil populations it is now clearer than ever the importance of understanding patient-specific neutrophil heterogeneity. As more neutrophil specific therapies are developed and tested in combination with immune checkpoint immunotherapies, it will be critical to understand how these drugs impact each neutrophil population and how the abundance of each one in the patients' tumours impacts therapy outcome.

## Neutrophil engineering: *ex vivo* reprogramming

Transfusion of exogenously-reprogrammed immune cells, such as CAR-T and CAR-NK therapies, are beginning to revolutionise how haematological cancers, such as lymphoma or adult and childhood leukaemias are treated. In these cancers, T and NK cells are manipulated to express recombinant chimeric antigen receptors (CARs), which significantly enhances their anti-tumour potential. However, to date, efficacy in solid tumours has been limited [92], although big efforts are made to improve this [93]. Moreover, clinical trials, based on preclinical studies in NOD scid γ (NSG) mice [94], are ongoing for advanced HCC with CAR-T cells targeting glypican-3, a cancer neoantigen widely expressed on the surface of the malignant hepatocytes [95]. Similarly, CAR-neutrophils are in the early stages of development and have shown promising preclinical results in glioblastoma [96]. This study genetically engineered human pluripotent stem cells (hPSCs) in order to create anti-glioblastoma CAR-neutrophils, which had enhanced anti-tumour activity both *in vitro* and *in vivo*. Mechanistically, the CAR-neutrophils were shown to be phenotypically similar to hPSC derived neutrophils but had greater anti-tumour functionality as assessed by neutrophil-tumour immune synapse formation, phagocytosis and ROS-mediated tumour killing (Figure 5) [96]. In line with this, we propose that CAR-neutrophils may be effective in HCC. Compared to CAR-T cells, neutrophils express matrix metalloproteinases which give them a superior ability to infiltrate liver tumours with a dense stroma. Furthermore, the short lifespan of CAR-neutrophils may reduce toxicities, a recurrent clinical issue experienced by patients receiving CAR-T therapy. In addition, neutrophils could also be genetically modified in order modulate specific pro-tumour and anti-tumour functions. To do so there is a unique pre-clinical model of immortalised myeloid progenitors the can be maintained, modified and expanded *ex vivo*, called Hoxb8-conditional myeloid progenitor cells. Neutrophils derived from this system are phenotypically similar to mouse primary neutrophils [97,98] and display neutrophil functions when administered *in vivo* (Figure 5) [99]. Furthermore, given the success of combination therapies in preclinical models in reprogramming neutrophils *in vivo* to drive anti-tumour immunity, transfusion of exogenously-stimulated neutrophils may be sufficient to drive an anti-cancer response. As a proof of concept, preclinical work has shown that transfusion of immature neutrophils isolated from LPS-treated mice is sufficient to reactivate anti-tumour immunity in immunotherapy-resistant mouse models of NASH-HCC [39]. Currently, granulocyte infusion for the treatment of refractory neutropenic sepsis is the only approved neutrophil cellular therapy [100]. In theory, granulocytes used for this purpose could be polarised to an anti-tumour phenotype *ex vivo* prior to infusion in order to stimulate anti-tumour immune responses. A fast-acting stimulus which robustly polarises neutrophils to an anti-tumour phenotype whilst not over activating them would need to be identified. Moreover, granulocyte transfusions carry significant risk which would need to be carefully considered, especially when genetically modifying or stimulating them. The main immediate risks being febrile reactions and pulmonary toxicity [100].

## Conclusion and outlook

Interrogation of TAN biology has finally entered the era of scRNAseq allowing for the first time the analysis of neutrophil heterogeneity and the identification of TAN subtypes, many of which appear to be conserved across cancers. However, this is just the start, and this cutting-edge technology is not without its limitations. Firstly, scRNAseq read depth is much lower than that of conventional bulk RNAseq and, as such, only a superficial picture of the neutrophil transcriptome is provided [101]. When this is coupled with the low abundance of RNA and high abundance of RNAses present in neutrophils, caution must be taken when analysing and interpreting scRNAseq from TANs. Secondly, bioinformatical clustering of scRNAseq data can lead to over clustering, and the fallacy of affirming the existence of more neutrophil subtypes than may exist. Ultimately, future work needs to focus on identifying surface protein markers that will allow for functional analysis and validation of proposed TAN subtypes and enable the identification of a suitable homogeneous and universal nomenclature going forward.

From a therapeutic perspective, neutrophils have conventionally been regarded as promoting tumorigenesis. As a result, immunotherapeutic strategies targeting neutrophils in cancer have largely focused on inhibiting the recruitment of neutrophils to the tumour. Recent data, however, suggest that neutrophils in the HCC tumour microenvironment can be programmed with anti-tumour functions if provided with the right exogenous stimulus and could even overcome immunotherapy resistance in NASH-HCC patients. Finally, neutrophil-based cell therapies may provide an exciting new therapeutic avenue and an area of research for improving our understanding of neutrophil-mediated anti-tumour immunity and improving HCC immunotherapy outcomes. Altogether, recent developments in our understanding of neutrophil biology in cancer has shed light on the cellular heterogeneity and plasticity of a once considered ‘inert’ cell. Future work should aim to exploit these recent findings to boost the efficacy of existent treatments and lead to the development of new immunotherapies for patients with cancer.

## Summary

- Neutrophils are a heterogeneous population of cells that can display a range of phenotypes in homeostasis, disease and cancer.
- Tumour-associated neutrophil (TAN) subtypes functionally correlate between cancers.
- Neutrophils exhibit cellular plasticity and can undergo reprogramming in the HCC tumour microenvironment.
- Reprogrammed neutrophils, *in situ* or *ex vivo*, represent a viable anti-cancer target.

## Open Access

Open access for this article was enabled by the participation of Newcastle University in an all-inclusive *Read & Publish* agreement with Portland Press and the Biochemical Society under a transformative agreement with JISC.

## Abbreviations

Ab: Antibody
ADCC: Antibody-dependant cellular cytotoxicity
APOA2: Apolipoprotein A-II
ARG1: Arginase 1
BLT1: Leukotriene B_4_ receptor 1
CAF: Cancer-associated fibroblast
CAR: Chimeric antigen receptor
Cas9: CRISPR-associated protein 9
CCL4: C-C motif chemokine ligand 4
CD: Cluster of differentiation
cDC1: Conventional dendritic cell 1
c-MET: Tyrosine kinase MET
CRISPR: Clustered regularly interspaced short palindromic repeats
CTLA-4: Cytotoxic T-lymphocyte-associated protein 4
CXCL5: CXC motif chemokine ligand 5
CXCL8: CXC motif chemokine ligand 8
CXCR2: CXC motif chemokine receptor 2
C5a: Complement component C5a
C5AR1: Complement component C5a receptor 1
C5b: Complement component C5b
ECM: Extracellular matrix
ELL2: Elongation factor for RNA polymerase II 2
GEMM: Genetically engineered mouse model
GMP: Granulocyte-monocyte progenitor
HBV: Hepatitis B virus
HCC: Hepatocellular carcinoma
HCV: Hepatitis C virus
HDN: High-density neutrophil
HLA class II: Human leukocyte antigen class II
Hoxb8: Homeobox B8
hPSC: Human pluripotent stem cell
HSPC: Hematopoietic stem and progenitor cell
ICC: Intrahepatic cholangiocarcinoma
IFIT1: Interferon induced protein with tetratricopeptide repeats 1
IFN: Interferon
IL-6: Interleukin 6
ISG15: Interferon-stimulated gene 15
JAK2: Janus kinase 2
LAM: Lipid-associated macrophage
LAN: Lipid-associated neutrophil
LDN: Low-density neutrophil
LPS: Lipopolysaccharide
LTB4: Leukotriene B_4_
METTL1: tRNA (guanine-N(7)-)-methyltransferase
MHC: Major histocompatibility complex
MMP8: Matrix metalloproteinase 8
MMP9: Matrix metalloproteinase 9
NASH: Non-alcoholic steatohepatitis
NDN: Normal-density neutrophil
NE: Neutrophil elastase
NeP: Neutrophil progenitor
NET: Neutrophil extracellular trap
NK: Natural killer
NO: Nitric oxide
NSCLC: Non-small cell lung cancer
NSG: NOD scid γ
PDAC: Pancreatic ductal adenocarcinoma
PD-1: Programmed cell death protein 1
PD-L1: Programmed death-ligand 1
PGE_2_: Prostaglandin E_2_
PSGL-1: P-selectin glycoprotein ligand-1
PTGS2: Prostaglandin-endoperoxide synthase 2
ROS: Reactive oxigen species
scRNAseq: Single-cell RNA sequencing
SIRPα: Signal regulatory protein α
SPP1: Osteopontin 1
S100A12: S100 calcium-binding protein A12
TAM: Tumour-associated macrophage
TAN: tumour-associated neutrophil
TCR: T cell receptor
TME: Tumour microenvironment
TNF: Tumour necrosis factor
TNFR1: Tumour necrosis factor receptor 1
TXNIP: Thioredoxin interacting protein
VEGF: Vascular endothelial growth factor
VEGFR2: Vascular endothelial growth factor receptor 2
VISTA: V-type immunoglobulin domain-containing suppressor of T-cell activation
XCR1: X-C motif chemokine receptor 1
